# A Case Report of an Invasive Infantile Fibrosarcoma of the Forearm

**Published:** 2016-08-12

**Authors:** Amy C. Kite, Lora M. Rotstein, Jennifer L. Rhodes

**Affiliations:** Division of Plastic and Reconstructive Surgery, Department of Surgery, Virginia Commonwealth University Medical Center, Richmond

**Keywords:** infantile fibrosarcoma, forearm tumors, World Pediatric Project, hemangioma, molecular translocation t(12;15)

## DESCRIPTION

**Figure 1:**
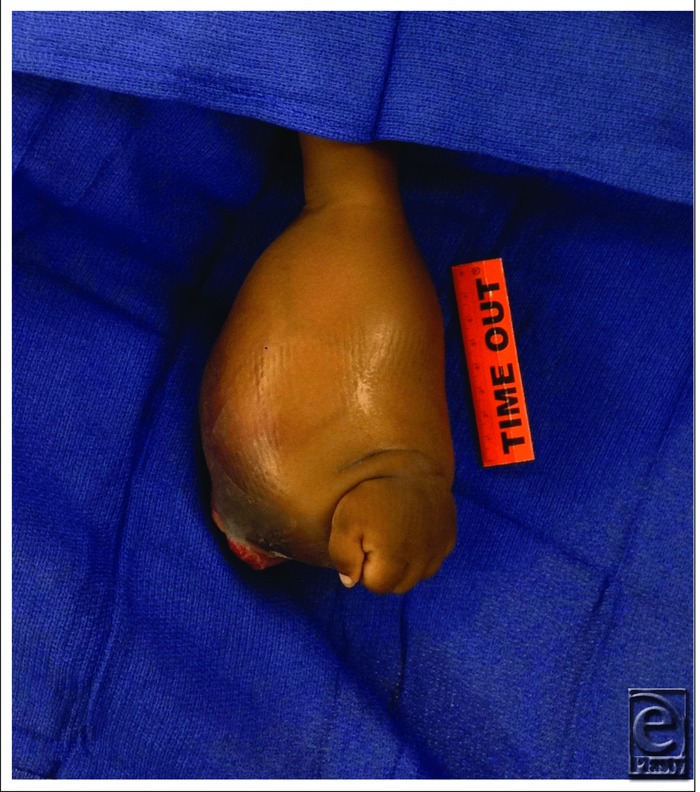
Dorsal view of left forearm infantile fibrosarcoma.

**Figure 2:**
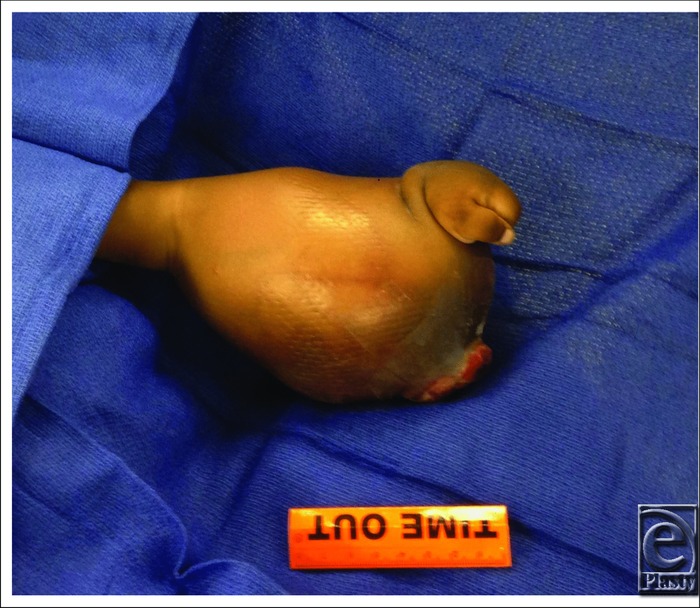
Radial view of tumor.

**Figure 3:**
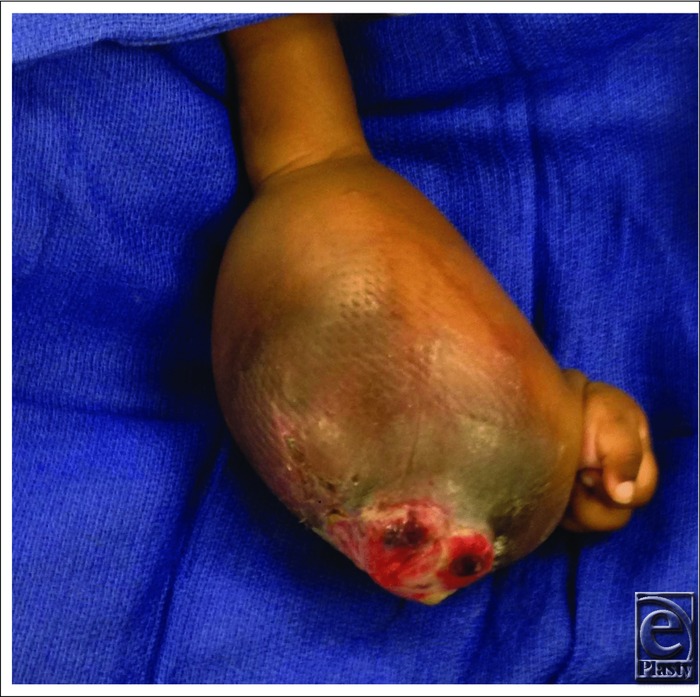
Ulceration present on volar left forearm infantile fibrosarcoma.

**Figure 4:**
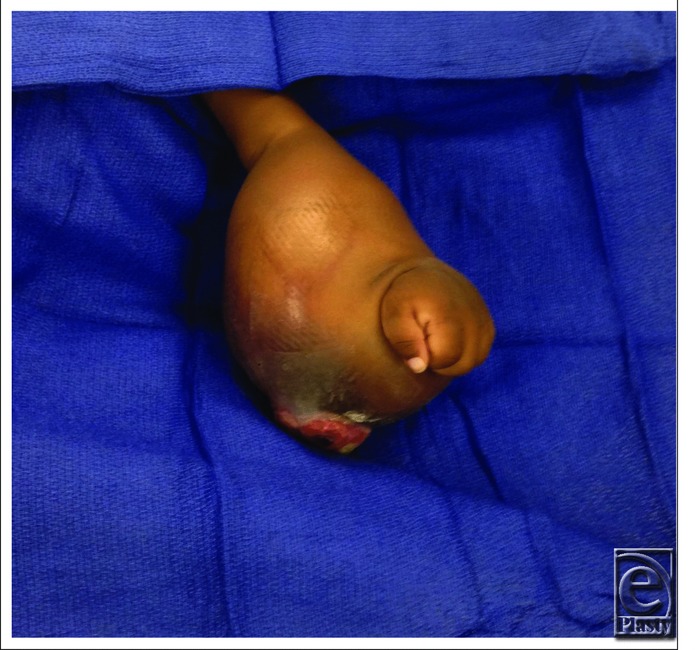
Infantile fibrosarcoma invading entirety of left forearm.

A 6-month-old female infant from St Vincent presented with a soft-tissue mass encompassing the left forearm (Figs 1–4). This was initially misdiagnosed as a hemangioma. Biopsy showed infantile fibrosarcoma, and the patient required a left transhumeral amputation due to the extensive involvement of the forearm.

## QUESTIONS

1. **What is infantile fibrosarcoma?**
2. **How do you diagnose infantile fibrosarcoma, and what are the important differential diagnoses you should consider?**
3. **What is the etiology of infantile fibrosarcoma?**
4. **What are the prognosis and treatment options available?**

## DISCUSSION

Infantile fibrosarcoma is a rare, malignant, highly vascularized, mesenchymal tumor that grows rapidly from soft tissues. Sarcomas comprise 7% of the malignancies seen in people younger than 20 years.^1^ Infantile fibrosarcoma accounts for 24% of the soft-tissue sarcomas seen in early infancy before 1 year of life.^1^ It often presents in the extremities, mostly occurring in the upper limb but also has been reported in the trunk, head and neck, and gastrointestinal tract.^2^ The tumor presents as a rapidly growing, poorly circumscribed, nontender mass. Our patient was referred by the World Pediatric Project, with a history of left forearm swelling at 3 month of age and gradual growth thereafter. She was initially evaluated by her pediatrician in St Vincent and sent to a pediatric surgeon in Barbados for further management. At that time, the mass was firm, tense, and diagnosed as a hemangioma. Propranolol was initiated for treatment, and the patient transferred to the United States for continued care.

Diagnosis can be difficult due to the appearance being similar to vascular malformations. If misdiagnosed and treated conservatively, this can be detrimental to the child, as there is a 7.3% mortality rate.^1^ The use of imaging modalities and biopsy is very important. Plain radiographs may show cortical thickening or destruction of bone, as these tumors can be very aggressive.^3^ Infantile fibrosarcoma appears as a vascular, heterogeneous, echogenic mass on the ultrasound scan. Since this alone would often be misdiagnosed as a vascular malformation, further tests are necessary. Magnetic resonance imaging is the imaging modality of choice that determines the extent of destruction of the lesion, helping guide resection.^3^^,^^4^ A magnetic resonance image was obtained in our patient, showing the mass extended proximally to the medial epicondyle and distally to the metacarpal heads. Biopsy is necessary, as imaging can still misdiagnose as hemangiomas, vascular malformations, rhabdomyosarcomas, malignant histiocytoma, and peripheral nerve sheath tumors.^5^ Immunohistochemistry further helps with diagnosis, as infantile fibrosarcoma is vimentin positive, S100 negative, and desmin negative.^3^ Histologically, infantile fibrosarcoma is similar to an adult fibrosarcoma, with densely packed uniform sheets of spindle cells in a herringbone pattern.^5^

The etiology of infantile fibrosarcoma remains unknown, but it is thought to arise secondary to molecular translocation. This translocation, t(12;15), results in the gene fusion product ETV6-NTRK3 and is detected with RT-PCR assays.^6^

Infantile fibrosarcoma has a much better prognosis than a fibrosarcoma in adults. There is a low rate of metastasis and lymph node involvement in younger children. Local recurrence is common, up to 43%; therefore, current treatment consists of wide local excision with negative margins.^1^ Chemotherapy or neoadjuvant chemotherapy may have a role in certain patients. Complete excision can be difficult due to the aggressive nature of this tumor, as it often invades muscle, bone, and neurovascular structures. Neoadjuvant chemotherapy can be used preoperatively for tumor shrinkage but is typically used in larger lesions in older children. Some recommend neoadjuvant chemotherapy to prevent amputation, as well as decrease local recurrence. If margins are positive after the initial resection, chemotherapy is recommended. The most common combination consists of vincristine, actinomycin D, and cyclophosphamide.^5^ Unfortunately, for the patient in this report, the tumor was too invasive for this therapy and amputation was the most effective treatment. It has been shown that 5-year disease-free survival is higher in patients who underwent both surgical excision and chemotherapy treatment.^7^ Therefore, treatment in the future may include not only resection but also chemotherapy to prevent local recurrence. Radiation can be an effective treatment option, although not often used because of the deleterious effects on growth plates and the development of secondary malignancies.^5^^,^^8^
